# Excessive Homeostatic Gain in Spinal Motoneurons in a Mouse Model of Amyotrophic Lateral Sclerosis

**DOI:** 10.1038/s41598-020-65685-8

**Published:** 2020-06-03

**Authors:** Su-Wei Kuo, Marc D. Binder, C. J. Heckman

**Affiliations:** 1 Department of Physiology, Northwestern University, Chicago, IL 60611 USA; 2 Department of Physiology & Biophysics, University of Washington School of Medicine, Seattle, WA 98195 USA; 3 Department of Physical Medicine and Rehabilitation, Northwestern University, Chicago, IL 60611 USA; 4 Department of Physical Therapy and Human Movement Sciences, Northwestern University, Chicago, IL 60611 USA

**Keywords:** Amyotrophic lateral sclerosis, Excitability

## Abstract

In the mSOD1 model of ALS, the excitability of motoneurons is poorly controlled, oscillating between hyperexcitable and hypoexcitable states during disease progression. The hyperexcitability is mediated by excessive activity of voltage-gated Na^+^ and Ca^2+^ channels that is initially counteracted by aberrant increases in cell size and conductance. The balance between these opposing actions collapses, however, at the time that the denervation of muscle fibers begins at about P50, resulting in a state of hypo-excitability and cell death. We propose that this process of neurodegeneration ensues from homeostatic dysregulation of excitability and have tested this hypothesis by perturbing a signal transduction pathway that plays a major role in controlling biogenesis and cell size. Our 『homeostatic dysregulation hypothesis' predicted that neonatal mSOD1 motoneurons would be much more sensitive to such perturbations than wild type controls and our results strongly support this hypothesis. Our results have important implications for therapeutic approaches to ALS.

## Introduction

Neurodegeneration, the progressive loss of the structural and functional integrity of neurons, is a hallmark of several neurological disorders including Parkinson's disease, Alzheimer's disease, Huntington's disease and amyotrophic lateral sclerosis (ALS). Although a host of disparate pathological processes have been associated with neurodegeneration, such as protein misfolding, mitochondrial dysfunction, disrupted axonal transport, membrane degeneration and apoptosis^1–3^, they all lead to a common physiological consequence, namely a failure of cellular homeostasis. One possibility is that this failure occurs because these mechanisms have been disabled and have insufficient power to respond to challenges. An alternative hypothesis is that the opposite occurs, namely that the sensitivity or gain of homeostatic regulation has become too high and thus, the cellular response to normal perturbations is excessive. A common problem with high-gain feedback systems is overcompensation resulting in increasing oscillations in the regulated variables^4^, and ultimately values that are incompatible with normal function.

In the work described here, we have explored the hypothesis that neurodegeneration ensues from homeostatic dysregulation of excitability. We focused on the spinal motoneuron, the major effector of the somatic motor system, whose high vulnerability to degeneration causes the principal symptomatology of ALS, an invariably lethal, adult-onset neurodegenerative disease. We monitored homeostasis at various time points during disease progression by measuring the most fundamental of motoneuron functions, the transduction of synaptic inputs into output spike trains, using a mouse model of ALS, the SOD1 mutant (mSOD1). If homeostatic mechanisms for excitability in ALS are impaired, we reason that the mutant motoneurons should exhibit a muted response to homeostatic challenges. In contrast, if the gain of homeostatic mechanisms for excitability in ALS has been rendered excessively high, then the mutant motoneurons should express excessive sensitivity to homeostatic challenges.

Our hypothesis was prompted by a review of previous studies of changes in excitability in motoneurons in mouse models of ALS, including our own, that suggested that these cells exhibit excessive homeostatic gain during disease progression. In the mSOD1 mouse model, there is a poorly controlled surge in excitability when the animals are still embryonic^5–7^. This surge is induced by excessive activity of voltage-gated Na^+^ and Ca^2+^ channels that induce alterations in anatomy^7^ and persistent inward currents (PICs)^8^ within in the motoneurons. As the animals mature (P0-P40), this surge in excitability mediated by enhanced PICs continues^9^, but is brought under control by the young adult state, probably by balancing increased PICs against an equally aberrant increase in cell size and electrical conductance^10–15^. This parallel, yet balanced increase in PICs and conductance may collapse at the time that the denervation of muscle fibers begins at about P50 as hypoexcitability develops^16^. Thus, there appears to be an oscillation of excessive homeostatic responses to perturbations, a transient and short-lived return to normalcy mediated by the balancing of opposing excesses, followed ultimately by complete failure of homeostasis leading to cell death.

To achieve a direct test of the sensitivity of homeostatic regulatory systems in the motoneurons of mSOD1 mice, we set out to perturb a signal transduction pathway that plays a major role in controlling biogenesis and cell size. To identify an appropriate target, we examined the canonical biogenesis pathway involving the target of rapamycin in mammals (mTOR) in protein interaction databases to identify interactions between proteins in the mTOR pathway and proteins linked to ALS. Our inquiries identified S6K, an enzyme that is downstream of mTOR, as a good candidate. We proceeded by utilizing a recently developed inhibitor (PF-4708671 or PF-47) of S6K to perturb phosphorylation of S6 in neonatal mice^17,18^. Our 『homeostatic dysregulation hypothesis' for neurodegeneration predicted that neonatal mSOD1 motoneurons would be more sensitive to PF-47 than wild-type controls. Our results strongly supported this hypothesis, revealing not only a marked reduction in cell size in mSOD1 motoneurons treated with PF-47, without significant effects in controls, but also equally large reductions in their conductance and PICs. In some cases, these reductions were so large that the cells became completely inexcitable, strongly suggesting that the homeostatic gain in the mutant was rendered excessive and ultimately lethal.

## Results

### Targeting the S6K1 pathway in ALS

#### Identifying the target protein for alterations of motoneuron size

**Figure 1:**
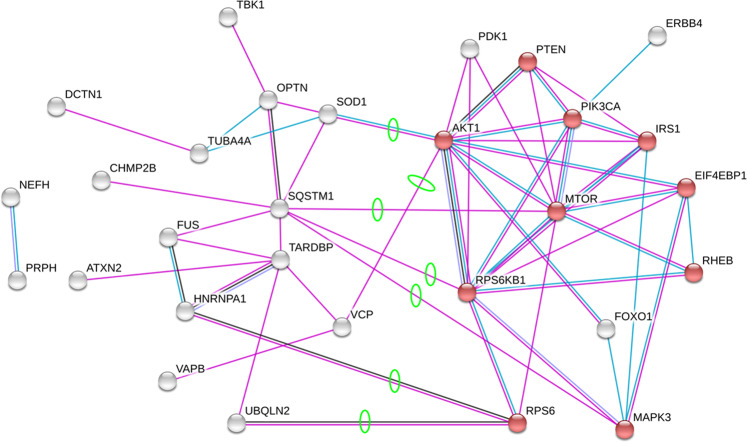
Visualization of protein-protein interactions between the mTOR pathway and ALS-associated proteins. Purple and blue lines represent the interactions from experiments and curated databases, respectively. The cluster at the right of this figure is formed by proteins in the mTOR pathway; the cluster to the left is formed by ALS-associated proteins. The solid red circles denote proteins with a 「Neuron Projection」 annotation in the GO database. Green circles highlight physical interactions between ALS-associated proteins and the mTOR pathway proteins. The analysis was performed on STRING database^68^.

To identify a potential target to manipulate motoneuron cell size, we began by considering signaling pathways that are causally related to biogenesis, with the rationale that the increase of cell size depends on the balance between biogenic and catabolic processes. The mTOR pathway is well-characterized for its regulatory role in cell growth/size by modulating protein synthesis and sensing external signals such as nutrient and growth factors^19–23^. Moreover the mTOR/AKT/S6K1 pathway is also extensively involved in neurodegenerative diseases, including ALS, through apoptosis/survival pathway regulation^24–26^. We hypothesized that the mTOR pathway might be directly involved in ALS pathology via its effect on cell growth in addition to its regulatory role in apoptosis. Thus, we sought to identify proteins in the mTOR pathway that physically interact with ALS-associated proteins in order to reveal candidate proteins for manipulation of protein homeostasis in ALS motoneurons. To accomplish this, we used STRING, a protein-protein interaction database. For our inputs to STRING, we used two sets of proteins, those in the mTOR pathway and those related to ALS (see *Methods*)^27–31^. The list of protein-protein interactions that emerged are presented in Table 1 and visualized in Fig. 1, where proteins that are highly expressed in neurons are highlighted in red (see the GO database^32^). When the mTOR pathway and ALS-associated proteins were clustered into two groups, the network connections revealed several protein - protein interactions between the mTOR and ALS pathways, including the following seven protein-protein pairs (for consistency with STRING, each protein is referred to by its encoding gene): *mTOR* - *SQSTM1*, *AKT* - *SOD1*, *AKT* - *VCP*, *MAPK3*/*ERK1* - *SQSTM1*, *RPS6* - *HNRNPA1, RPS6* - *UBQLN2* and *RPS6KB1* - *SQSTM1* (Table 1).

**Table 1:** Protein-protein interaction between mTOR pathway and ALS.

ALS-associated proteins^†^		Proteins in the mTOR pathway^†^	ALS x mTOR pathway protein interactions		
			Protein-protein interactions^†^		Value*
*ALS2*	*NEFH*	*AKT1*	*AKT1*	*SOD1*	0.925
*ALS3*	*OPTN*	*EIF4EBP1*	*AKT1*	*VCP*	0.727
*ALS7*	*PFN1*	*FOXO1*	*MAPK3/ERK1*	*SQSTM1*	0.623
*ALSDC*	*PRPH*	*IRS1*	*MTOR*	*SQSTM1*	0.573
*ANG*	*SETX*	*mTOR*	*RPS6*	*HNRNPA1*	0.591
*ATXN2*	*SIGMAR1*	*MAPK3/ERK1*	*RPS6*	*UBQLN2*	0.453
*C9ORF72*	*SOD1*	*PI3K*	*RPS6KB1*	*SQSTM1*	0.573
*CHCHD10*	*SPG11*	*PTEN*			
*CHMP2B*	*SQSTM1*	*PDK1*			
*DCTN1*	*TARDBP*	*RPS6KB1*			
*ERBB4*	*TBK1*	*RPS6*			
*FIG4*	*TUBA4A*	*RHEB*			
*FUS*	*UBQLN2*				
*HNRNPA1*	*VAPB*				
*MATR3*	*VCP*				

^*^The interaction value is determined by integrated probabilities of interactions from various types of evidence channels^69^.

^†^All proteins presented in the Fig. 1 and Table 1 use gene nomenclature from the Uniprot database^70^.

In this list of seven interactions, five genes associate with the mTOR pathway: *mTOR, AKT, MAPK3/ERK1, RPS6 and RPSKB1*. We eliminated *MAPK3/ERK1* because currently there has been no strong evidence to show its involvement in ALS from either clinical cases or animal model^33–37^. In contrast, *AKT* and *mTOR* have been previously studied for treating ALS^26,38^. The administration of rapamycin, an mTOR inhibitor, showed an augmentation of ALS disease progression^39^, whereas increased phosphorylation levels of AKT promoted survival in the hG93A-SOD1 model^26,40^. We thus had concerns whether actions of AKT and mTOR might have many other effects in addition to potentially altering cell size. On the other hand, *RPS6* encodes the protein RPS6 while *RPS6K1* encodes S6K1, a specific isoform of S6K. S6K1 is known to play a major role in regulating protein translation and has been shown to play a direct and important role in regulating cell size^17,41^. Moreover, S6K1 expression level is elevated in both mSOD1 mice and ALS patients^42–44^. Thus, we decided to target S6K1 to perturb cellular biogenesis.

#### Optimization of S6K1 inhibitor, PF-4708671

Recently, a specific inhibitor of S6K1 phosphorylation has been developed, PF-4708671^17^, with effective penetration into the CNS^18^. At the outset of our research, however, no toxicology or pharmacokinetics/pharmacodynamics reports were available for the administration of PF-4708671 to neonatal mice. Therefore, we conducted our own trials by administrating 20 mM PF-4708671 to five litters of wild-type mice at P2-P8 through IP injection at strengths of 10, 30 or 60 mg/kg per day with a littermate vehicle control group to evaluate inhibitor toxicity and effective dosage.

The 60 mg/kg per day groups showed significantly increased mortality rate starting from P4 and much lower overall survival rate at P8 (*P* < 0.05, Pairwise Comparisons). In contrast, the 10 and 30 mg/kg per day groups and the vehicle control group showed no change in mortality rate. As a result, we decided to test the efficacy of PF-4708671 at the 30 mg/kg per day dose in regulating cell size as evaluated by using two-photon fluorescent microscopy.

### Lumbar spinal motoneuron hypertrophy in neonate hG93A-SOD1 ALS mice

#### Increased hG93A-SOD1 motoneuron soma volume

Although we have clearly established that motoneuron input conductance is sharply increased in neonatal hG93A-SOD1 mice^12^, a corresponding increase in cell size has only been demonstrated in neonatal hG85R-SOD1 motoneurons^10,11^. To determine whether or not the hG93A-SOD1 motoneurons also undergo neonatal hypertrophy, the wild-type and hG93A-SOD1 mice were sacrificed between P8 and P10, and the ventrolateral region of lumbar spinal cord was examined using two-photon fluorescent microscopy. P8-P10 chosen because myelination begins at P11-P12^45,46^, which obscures the imaging and thus compromises precise measurements of soma volume.

**Figure 2:**
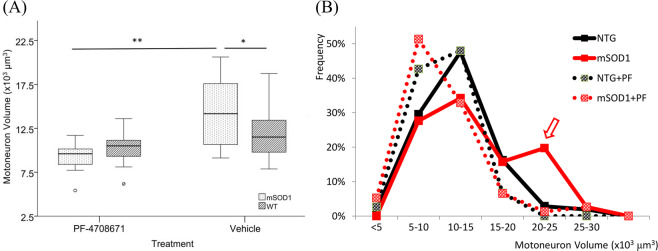
Effects of PF-4608671 administration of motoneuron size. **(A**) Motoneuron volume is significantly larger in hG93A-SOD1 group compared to wild-type control. The administration of PF-4608671 significantly reduced motoneuron soma volume in hG93A-SOD1. (*P < 0.05; **P < 0.01; WT, *N* = 120/24 [# of MNs/animals]; mSOD1, *N* = 60/12; WT + PF, *N* = 75/15; mSOD1+PF, *N* = 76/15). (**B**) The soma volume measures are binned every 5,000 μm^3^. The percentage is calculated as: (number of motoneurons in specific bin/number of total motoneurons in specific group). Increased frequency in large motoneurons and decreased frequency in medium motoneurons with less affected small motoneurons in hG93A-SOD1 group indicate the increase in volume of certain medium motoneurons. The application of PF-4708671 successfully restored the two-peak pattern in hG93A-SOD1 back to single peak as wild-type. Wild-type, WT; hG93A-SOD1, mSOD1; PF-4708671, PF; MN, motoneuron.

We measured motoneuron soma volume from three-dimensional scanned images and found a significant increase in the hG93A-SOD1 animals compared to the wild-type littermate controls (14156.13 ± 3920.44 μm^3^ vs. 11812.62 ± 2653.22 μm^3^; *P* < 0.05, one-way ANOVA; Fig. 2A). Our measurements also revealed that the increase in average soma size in hG93A-SOD1 motoneurons was non-uniform across the motoneuron pools. Figure 2B shows that the distribution of soma sizes had two peaks, one centered in the normal range and matching wild-type controls, and one peak at the large end of the range where few wild-type cells were present (comparative distributions are indicated using solid black and red lines). This result suggests that although hG93A-SOD1 motoneurons included many cells with normal cell volumes, a subset became abnormally large.

#### PF-4708671 prevents hG93A-SOD1 motoneuron enlargement

Given the hypertrophy in soma volume established above, we decided to test whether inhibiting S6K1 by PF-4708671 could prevent or reduce the abnormal growth of SOD1 mutant motoneurons. After seven days of administration of PF (30 mg/kg each day from P2-P8) in both wild-type and hG93A-SOD1 mice, we found a significant reduction in motoneuron soma volume in the hG93A-SOD1 receiving PF-4708671 compared to untreated hG93A-SOD1 group (9348.94.66 ± 1613.1 μm^3^ vs. 14156.13 ± 3920.44 μm^3^; *P* < 0.001, Pairwise Comparisons). The treated wild-type mice also showed some degree of soma volume reduction, but it was much less than that seen in the hG93A-SOD1 group (10293.22 ± 1757.85 vs. 11812.62 ± 2653.22 μm^3^). Because of the differential responses to S6K1 inhibition, the average soma volume of both PF-4708671 treated wild-type and hG93A-SOD1 groups are at similar levels (10293.22 ± 1757.85 μm^3^ vs. 9985.66 ± 1613.1 μm^3^). This differential effect suggests that the homeostatic systems in hG93A-SOD1 motoneurons are more sensitive to actions of PF-4708671 than that of wild-type motoneurons.

The administration of PF-4708671, however, did not equally affect all sizes of hG93A-SOD1 motoneurons. Figure 2B shows that PF-4708671 eliminated the peak of large cells in the untreated hG93A-SOD1 motoneurons (compare solid red to dotted red distributions) (Pearson χ^2^ = 56.259, *P* < 0.01). In effect, PF-4708671 prevented the generation of extremely large hG93A-SOD1 motoneurons, resulting in a unimodal size distribution with a similar range to that observed in the wild-type control mice. This result suggests the following: hG93A-SOD1 motoneurons that undergo abnormal enlargement are those with medium to large somata, whereas small motoneurons in both wild-type and hG93A-SOD1 are less affected by PF-470867. Moreover the medium to large motoneurons in wild-type animals were also relatively insensitive [cumulative percentage of motoneurons >15,000 μm^3^, a 14.3% reduction from 21% (wild-type) to 6.7% (wild-type + PF-4708671)]. This implies that the medium to large hG93A-SOD1 motoneurons are most sensitive to S6K1 inhibitor disruption [for motoneurons with volumes > 15,000 μm^3^, there was 27.6% reduction from 38.1% (hG93A-SOD1) to 10.5% (hG93A-SOD1 + PF-4708671)].

### Abnormal electrical properties in neonate hG93A-SOD1 ALS mice

#### Altered electrical properties in hG93A-SOD1 motoneurons

**Figure 3:**
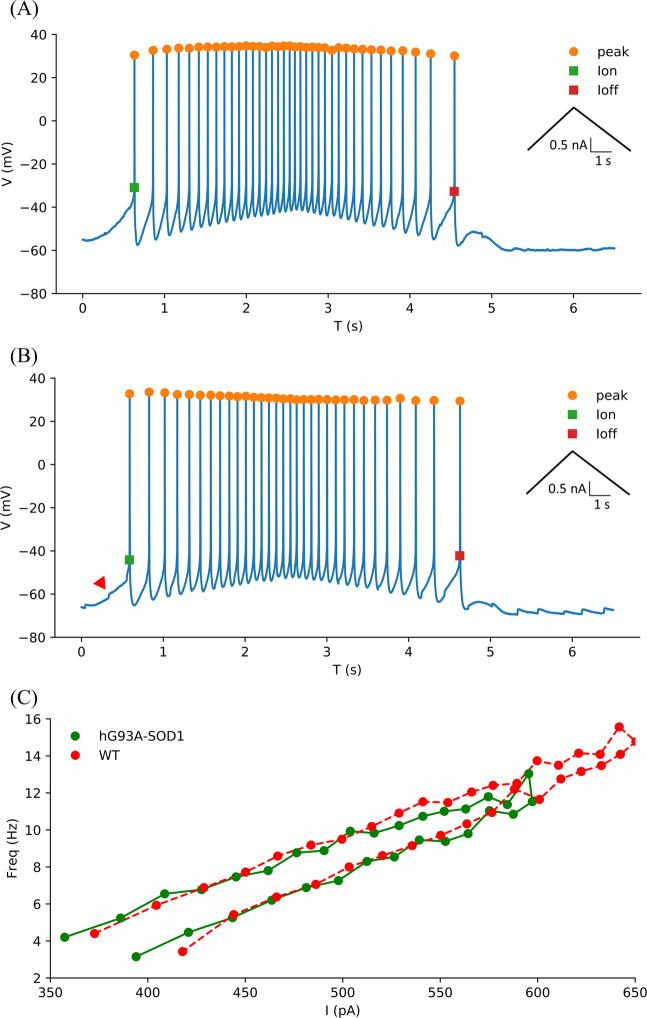
Comparison of motoneuron firing frequency-current relationships in hG93A-SOD1 and wild-type mice. (**A,B**) Repetitive firing in response to the injected current ramps in a representative wild-type motoneuron (**A**) and an hG93A-SOD1 motoneuron (**B**). The corresponding current amplitude at first spike onset and last spike are defined as Ion and Ioff and the difference as ΔI. C, The instantaneous frequency was calculated from (A) and (B) and plotted against the corresponding current intensity to generate the F-I relationships of the wild-type (red trace) and the hG93A-SOD1 (green trace), respectively. The red arrow in (B) indicates the onset of a PIC. The ascending and descending phases of the F-I relationships were then fitted with a linear equation to obtain the slopes, which are reported in Table 2 and used as a metric for excitability.

**Figure 4:**
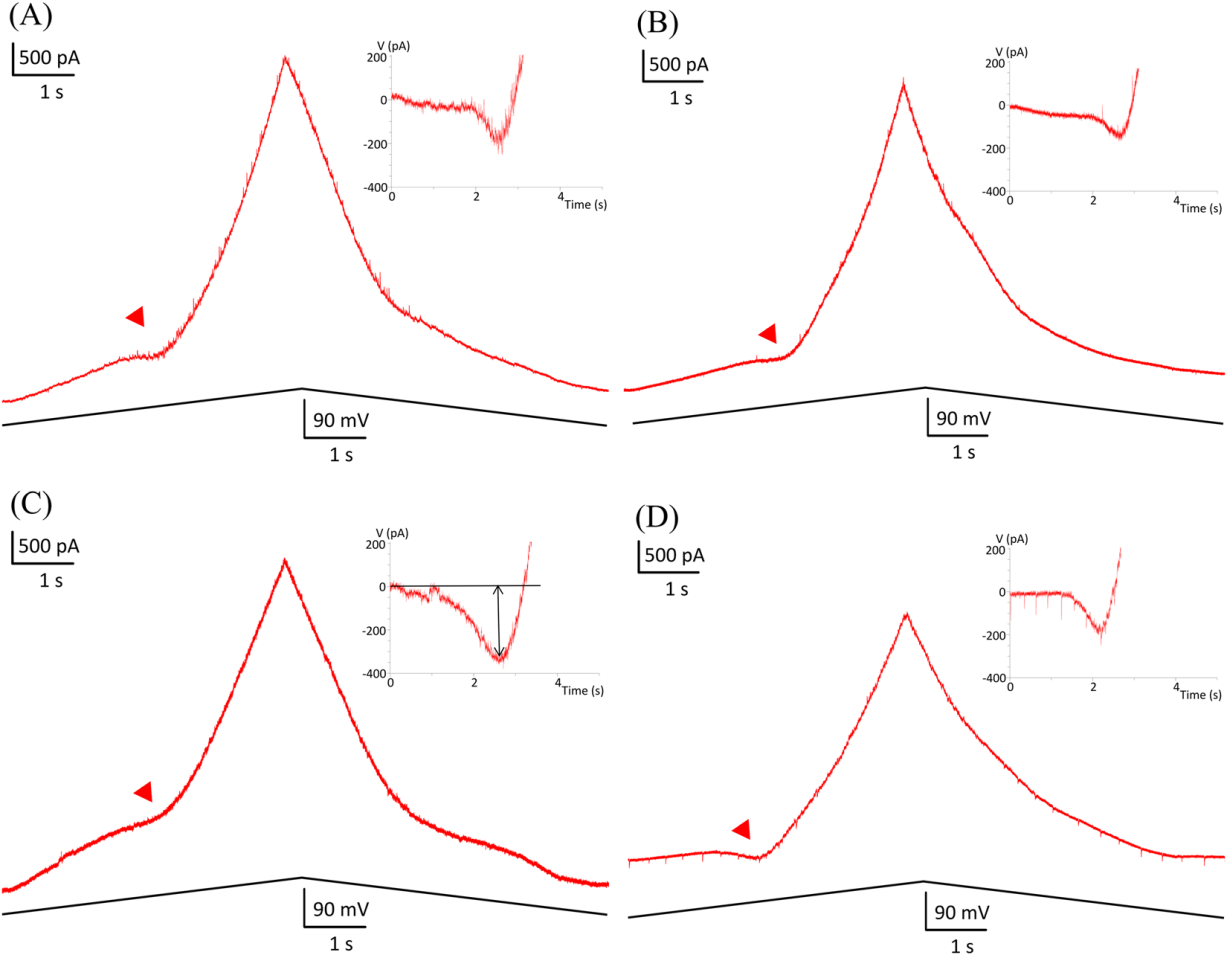
Comparison of persistent inward currents (PICs) in wild-type and hG93A-SOD1 motoneurons. **A** and **C**, PICs induced by slow, voltage bi-ramps (20 mV/s, from −80 mV to +10 mV) are indicated by the red arrows. The hG93A-SOD1 motoneurons (**C**) showed significantly larger PICs than the wild-types (**A**). The current response was then subtracted by leak current calculated before PIC happened as shown by linear fitting line, the black solid line in inlet of (**C**). **B** and **D**, for wild-type (**B**) and hG93A-SOD1 (**D**) received PF-4708671, they showed similar PIC amplitude as in (**A**). The average amplitudes of PIC of wild-type and hG93A-SOD1 were reported in Table 2.

We next replicated the studies of Quinlan *et al*.^47^ that showed an increase in input conductances and PICs in neonatal hG93A-SOD1motoneurons to provide a basis for determining whether the effects of PF-4708671 in reducing cell size also affected motoneuron electrical properties. To do so, we made whole-cell patch clamp recordings from ventrolateral motoneurons in the lumbar spinal cord slices. Two examples of F-I functions generated by a linearly increasing and decreasing injected current are shown in Fig. 3. We found no differences for the threshold and slope parameters of the F-I relationships between the hG93A-SOD1 and control motoneurons (see Table 2), suggested no net change in overall electrical excitability. We then assessed both input conductances and PIC amplitudes under voltage clamp, using linearly rising and falling voltage commands, as illustrated in Fig. 4. Both the PIC amplitudes (−357 ± 111 pA vs. −200 ± 73 pA, *P* < 0.01, Pairwise Comparisons,) and the input conductances (32 ± 10 nS vs. 24 ± 10 nS, *P* < 0.01, Pairwise Comparisons) were significantly larger in the hG93A-SOD1 motoneurons compared to control cells. Therefore, as previously shown by Quinlan and colleagues^12^, the increased input conductance seems to be counteracted by enhanced PIC amplitudes to effectively stabilize excitability, at least as indicated by F-I relations.

**Table 2:** Electrical properties of motoneurons.

	WT (N=25)	mSOD1 (N=22)	WT+PF (N=21)	mSOD1+PF (N = 24)	
				Non-hypoexcitable (N = 20)	Hypoexcitable (N = 4)
G_in_ (nS)	24 ± 10	32 ± 10 **(WT)	22 ± 10	24 ± 8 **(mSOD1)	29 ± 10
RMP (mV)	−59 ± 4	−60 ± 5	−58 ± 5	−60 ± 6	−59 ± 3
I_on_ (pA)	445 ± 247	466 ± 346	417 ± 232	468 ± 307	—
I_off_ (pA)	508 ± 291	521 ± 382	511 ± 280	547 ± 352	—
ΔI (pA)	63 ± 109	55 ± 97	94 ± 137	79 ± 152	—
F-I_Asd_ (Hz/nA)	34 ± 18	36 ± 23	37 ± 25	38 ± 27	—
F-I_Dsd_ (Hz/nA)	36 ± 19	39 ± 26	39 ± 26	41 ± 25	—
AP overshoot (mV)	29 ± 10	30 ± 11	30 ± 9	29 ± 10	27 ± 6
Voltage threshold (mV)	−30 ± 6	−29 ± 5	−30 ± 7	−28 ± 6	−28 ± 4
Capacitance (pF)	332 ± 103	338 ± 112	334 ± 107	331 ± 114	345 ± 64
PIC					
Amplitude (pA)	−200 ± 73	−357 ± 111 **(WT)	−202 ± 91	−229 ± 79 **(mSOD1)	−87 ± 29 **(mSOD1+PF)
Peak voltage(mV)	−21 ± 6	−21 ± 7	−22 ± 9	−23 ± 6	−27 ± 5
Onset voltage(mV)	−46 ± 7	−45 ± 6	−47 ± 8	−47 ± 6	−47 ± 4

*P*-values were calculated by Turkey's test in *post-hoc* analysis in comparison to group as specified (**P<0.01). Wild-type, WT; hG93A-SOD1, mSOD1; PF-4708671, PF; input conductance, G_in_; persistent inward current, PIC; Ion, onset current in F-I relationship; Ioff, offset current in F-I relationship; ΔI, difference between Ion and Ioff; F-IAsd, slope of linear fit in ascending phase of F-I relationship; F-IDsd, slope of linear fit in descending phase of F-I relationship.

#### PF-4708671 reversed input conductance and PIC changes in hG93A-SOD1

To determine whether the reduction in cell size by PF-4708671 induces concomitant changes in the electrical properties of hG93A-SOD1 motoneurons, we performed a second series of IP injections of this agent in wild-type and hG93A-SOD1 mice from P2-P6, and prepared the mice for slice patch-clamp on P7-P9. The aberrant increase in conductances and PICs previously showed in hG93A-SOD1 group were both significantly reduced in the hG93A-SOD1 motoneurons that received the PF-4708671 treatment [G_i_: 32 ± 10 nS vs. 24 ± 8 nS, *P* < 0.01, Pairwise Comparisons; PIC: −357 ± 111 pA vs. −229 ± 79 pA, *P* < 0.01, Pairwise Comparisons; Table 2]. More importantly, the net excitability as indicated by ascending and descending F-I relationship (F-I_Asd_ and F-I_Dsd_) still remained unchanged (Table 2), suggesting a possible intrinsic mechanism in maintaining excitability, i.e., homeostasis. The maintained overall excitability, with concomitant changes in input conductance and PIC in the hG93A-SOD1 with PF-4708671 suggests that both parameters are under homeostatic control as cell size was reduced, resulting in maintained excitability. Two further points are salient: The application of PF-4708671 did not affect other electrical properties such as resting membrane potential, or the voltages of PIC peak and onset (Table 2), and, in control cells, the lack of effect of PF-4708671 on soma sizes was matched by a lack of significant effects on their conductances and PICs (Table 2). Thus, these results point to a higher sensitivity of homeostatic responses in hG93A-SOD1 motoneurons than in controls.

#### Hypoexcitable motoneurons in hG93A-SOD1 mice that received PF-4708671

**Figure 5:**
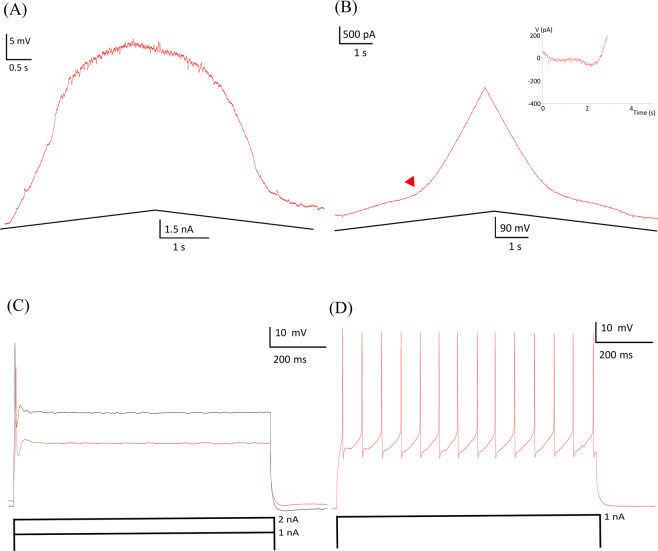
Example of a hypoexcitable hG93A-SOD1 motoneuron. **(A**) Hypoexcitable hG93A-SOD1 motoneurons cannot sustain repetitive firing in response to slow injected current ramps (0.6 nA/s). (**B**) The PIC from the same motoneuron was significantly smaller than those in hG93A-SOD1 motoneurons that are capable of repetitive firing. (**C**) This hypoexcitable motoneuron could generate only a single action potential (red trace) during a prolonged (800 ms) 1 nA depolarizing current step, and stayed hypoexcitable (black trace) even when input current was doubled to 2 nA. (**D**) In contrast to hypoexcitable motoneurons, wild-type motoneurons can fire repetitively under 1 nA, 800 ms depolarizing current step.

Of the 24 motoneurons in Table 2 that were from hG93A-SOD1 + PF-4708671 treatment group, 4 were found to exhibit hypoexcitable behavior (Fig. 5). These hypoexcitable hG93A-SOD1 + PF-4708671 motoneurons displayed abnormal responses to slow current ramps even when the peak amplitude of command current ramp was doubled. They either failed to fire entirely or were only able to fire a few spikes. These hypoexcitable cells were, however, capable of generating a single action potential in response to a short current step. This level of hypoexcitability was not seen in any of the control motoneurons.

**Figure 6:**
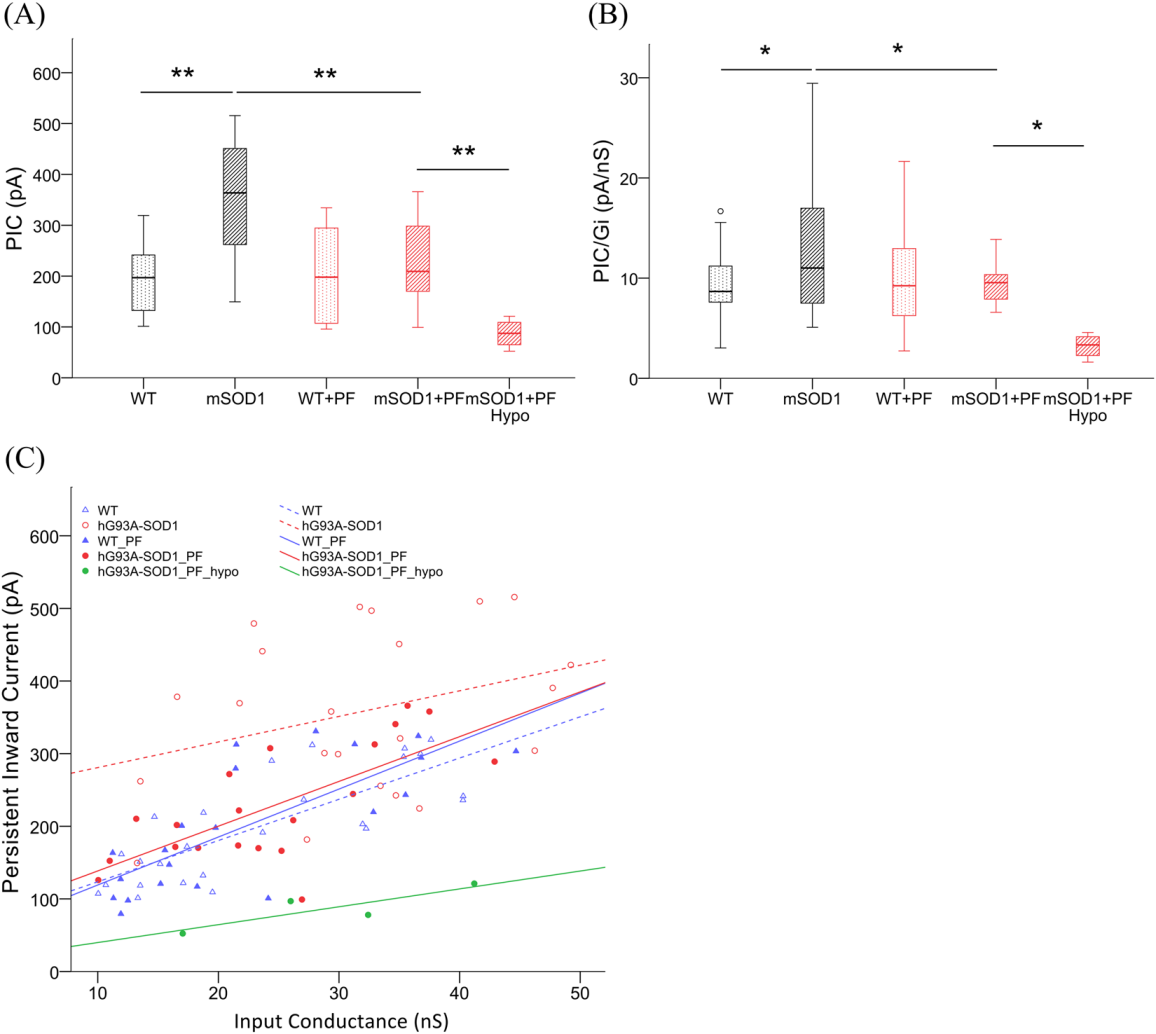
PF-4708671 treatment prevents PIC enhancement in hG93A-SOD1 motoneurons. **(A**) PICs were significantly larger in hG93A-SOD1 motoneurons compared to WT, but not in those cells that received PF-4708671. Four out of twenty-four (17%) hG93A-SOD1 motoneurons that received PF-4708671 showed profound hypoexcitability and much smaller PICs than other motoneurons, but no significant change in input conductance (G_in_). (**B**) The ratio of PIC to G_in_ was taken as a metric of altered excitability. The result showed similar trend and significant changes as in PIC analysis in (**A**). (**C**) To further investigate the relationship between PIC and input conductance, we plotted their distributions and fitted the data with a linear equation. Within a comparable input conductance range, the hG93A-SOD1 motoneurons had larger PICs. In contrast, the hG93A-SOD1 motoneurons in animals treated with PF-4708671, had smaller PICs regardless of G_in_. Wild-type, WT; 30 mg/kgBW PF-4708671, PF; hypoexcitable, Hypo; persistent inward current, PIC; input conductance, G_in_; **P < 0.01.

Previous studies have demonstrated that the Na^+^ component of the PIC is essential for initiation of spikes during steady and slowly rising inputs^48–50^. Consistent with this finding, average PIC amplitudes from these four hypoexcitable motoneurons were much smaller than those from the rest of the hG93A-SOD1motoneurons (*N* = 20) that received PF-4708671 (see the example in Figs. 5B, and 6A and Table 2). Despite this small sample size, this difference was significant (−87 ± 29 pA vs. −229 ± 79 pA, *P* < 0.01, Pairwise Comparisons). Input conductance, resting membrane potential and action potential threshold were not significantly different in these hypo-excitable cells compared to controls (Table 2). Consistent with these results, when we analyzed the ratio of PIC to input conductance (PIC/G_i_), we found that these hypoexcitable motoneurons had significantly smaller ratios, suggesting that in at least a subset of hG93A-SOD1 cells, the homeostatic gain was so high that over compensation resulted in excessively smaller PICs (Fig. 6B).

To further scrutinize the relationship between PIC and input conductance, the data were plotted and fit by linear regression (Fig. 6C). Generally speaking, the higher the conductance of a motoneuron, the larger the PIC. This relationship was weakest for the hG93A-SOD1 motoneurons, but it was notable that treatment with PF-4708671 fully restored the relationship seen in wild-type motoneurons whereas PF-4708671 had no significant impact on this relation in the wild-type cells (*P* > 0.05). The strength of the relationship between PIC and conductance for the four hypoexcitable cells was well below that of all the other cells.

## Discussion

The underlying premise of the present study is that during their life course, from development through senescence, neurons regulate their excitability through a host of homeostatic control mechanisms to maintain a firing rate within their optimal operational range^51,52^ and that dysregulation of any of these homeostatic mechanisms produced by innate aberrancies may lead to premature cell death. Our review of previous studies on the morbidity of motoneurons in the mSOD1 mouse model of ALS strongly suggested that these cells are afflicted with a deleterious 『high gain' in the homeostatic systems controlling their excitability. Embryonic mSOD1 motoneurons display a poorly controlled surge in excitability induced by excessive activity of the voltage-gated Na^+^ and Ca^2+^ channels that mediate persistent inward currents (PICs)^8^. As the mutant animals mature (P0-P40), this surge in excitability mediated by enhanced PICs continues, but it is effectively counteracted by an equally aberrant increase in cell size and electrical conductance^10,12^. There appears to be a limit, however, to the viability of this strategy as it is the largest motoneurons that are the first to degenerate with disease progression^15,53–55^. This finding suggests that the parallel, but balanced increase PICs and conductance collapses at the time that the denervation of muscle fibers begins at about P50, resulting in emergence of a state of hypo-excitability^14^. Thus, the life course of the disease is characterized by a repetition of excessive homeostatic responses to perturbations that ultimately lead to cell death.

The results reported here only apply to the specific mouse model that we have studied, the high expressor G93A mSOD1 mouse^56^. Nonetheless, early alterations in the excitability of motoneurons similar to those we have observed in this G93A model have also been demonstrated in several different ALS models, including G85R mSOD1 mice^57,58^, and cells derived from human patients^59^. In addition, the transition from hyper- to hypo-excitability seen in adult G93A mice^14^ has been shown to occur with time in patient derived cells^60–62^. Dysregulation of homeostatic plasticity is also consistent with the wide spread disruptions in cellular systems in neurodegenerative diseases including protein misfolding, mitochondrial dysfunction, altered endoplasmic reticulum function, disrupted axonal transport, membrane degeneration and apoptosis^1–3^. Moreover, the meta-analysis performed on the extant literature by Mitchell and colleagues^63,64^ emphasizes the role of homeostatic dysregulation and revealed a striking pattern of growing oscillations in multiple cellular functions during the life span of mouse ALS models, exactly what would be expected from homeostatic systems that over-compensate for perturbations

The results reported here indicate that suppressing S6K1 by administration PF-4708671 prevents motoneuron hypertrophy as well as the accompanying increased input conductance and PIC amplitude that are characteristic of the pathological changes in mSOD1 motoneurons during disease progression. The much greater effect of PF-4708671 on size and electrical properties of hG93A-SOD1 motoneurons than of controls suggests that other signal transduction pathways are not able to compensate for the suppression of S6K1 activities. It may well be that these other signal transduction pathways are also impaired in hSOD1-G93A mice. The high gain of the homeostatic systems of the mSOD1 motoneurons in response to PF-4708671 treatment initially restored soma volume to normal values accompanied by proportional changes in the electrical properties of these cells. Further examination of the data, however, revealed that this apparently beneficial effect of high gain homeostasis at this very young age already showed signs of being excessive, as a hypoexcitable subpopulation of cells emerged with particularly small PICs. In the absence of treatment with PF-4708671, mSOD1 motoneurons do not become hypoexcitabile until the onset of denervation at around P50^16^.

An interesting feature of the soma volume data is that, before administration of PF47, the increased mean volume appears to be primarily driven by increases at the large end of the spectrum (Fig. 2B). These large cells might represent motoneurons of the fast fatigable (FF) type, which are the first to become hypoexcitable and the first to degenerate as the disease progresses^53,54^. This point is speculative, as we cannot differentiate motoneuron type in this data set at this early stage, though there are clear differences between slow and fast motoneurons^13^. The increase in cell size, conductance and PICs seems likely to be a source of cellular stress. As yet, PICs have not been measured as the disease progresses into the adult state, but input conductance is still elevated in the young adult state^14^. Anatomical parameters parallel this increase, with increased soma diameter and increased dendritic length persisting until the disease develops, up to the point at which both trends reverse, resulting in smaller cells with shorter dendrites^15,55^. In large part, this reversal may reflect the degeneration of large FF cells, though as Dukappiti *et al*. point out, there appears to be actual shrinkage of the surviving cells^15^. Overall these trends are consistent with hypervigilant homeostasis causing excessive increases in cell sizes and PIC amplitudes, resulting in stress that contributes to the subsequent collapse. As type FF motoneurons are already the largest, this mechanism is consistent with their early demise compared to FR and S motoneurons.

Our results have important implications for therapeutic approaches to ALS. Consider, for example, riluzole, which has been shown to exert a modest effect in prolonging life in both humans with ALS and animal models^65^. At the concentration achievable by oral administration, riluzole reduces glutamate release and inhibits Na^+^ channel activation, with a clear effect on the Na^+^ component of the PIC measured in the present studies^65^. If homeostatic gain is excessive, initial treatment with riluzole may have a strong beneficial action. Motoneurons may, however, develop excessive responses within a relatively short time frame, as suggested by our previous study in cultured motoneurons^66^, thus limiting riluzole's effectiveness. The same consideration applies to a drug like PF47 that affects cell size. It likely should be given early, while sizes are still excessive, but prolonged administration may be counterproductive. Once the time course and nature of this homeostatic dysregulation is more fully understood it may be possible to devise a more effective dosing schedule, perhaps one in which the therapeutic agent is given for short periods interspersed with recovery periods. Further study of motoneuron responses to perturbations of multiple cellular systems will be essential in evaluating homoestatic dysregulation as a significant mechanism of ALS and guiding future therapies.

## Materials and Methods

### Animals and drug

#### Animals

All animal protocols and procedures were approved by Northwestern University Institutional Animal Care and Use Committee (IACUC). Mice were breed and maintained in accordance with NIH guidelines in specific-pathogen free barrier room within Northwestern University Center of Comparative Medicine (CCM) animal facility. The mice were maintained under a 12/12 light-dark cycle, with food and water provided *ad libitum*. We used the G93A SOD1 mouse model^56,67^. Wild-type female mice (B6.SJL-*Ptprc*^a^ *Pepc*^b^/BoyJ), homozygous female Hb9-GFP mice ((B6.Cg-Tg(Hlxb9-GFP)1Tmj/J) and male transgenic mice overexpressing hG93A-SOD1 (B6SJL-Tg(SOD1*G93A)1Gur/J) were all obtained from the Jackson Laboratory (Bar Harbor, ME, USA). Wild-type female mice were breed with hG93A-SOD1 male mice in a hemizygous manner, and tail-clips from pups were sent to Transnetyx® (Cordova, TN, USA) for hG93A-SOD1genotyping (SOD1: Forward Primer- CAG TAA CTG AGA GTT TAC CCT TTG GT; Reverse Primer- CAC ACT AAT GCT CTG GGA AGA AAG A). For the two-photon imaging experiments, homozygous Hb9-GFP female mice were mated with hG93A-SOD1 male mice, tissues were collected after two-photon imaging and genotyped by Transnetyx® (GFP Forward Primer- CGT CGT CCT TGA AGA AGA TGG T; Reverse Primer- CAC ATG AAG CAG CAC GAC TT). The hemizygote Hb9-GFP did not affect the GFP signal which was visible from P7 onward. For the electrophysiology experiments and behavior tests, only B6SJL background mice were used (wild-type female x hG93A-SOD1 male). hG93A-SOD1 mice with lower copy numbers were not used (signal strength <70 in Transnetyx® raw data). Non-transgenic littermate controls were used in all of the experiments.

#### PF-4708671 preparation

PF-4708671 (Tocris, Minneapolis, MN, USA), a S6K1specific inhibitor (Ki = 20 nM; IC50 = 160 nM)^17,18^, was dissolved in dimethyl sulfoxide (DMSO) (Sigma, St. Louis, MO, USA) and aliquots were kept at −20 °C. Dissolved PF-4708671 was diluted to 20 mM by 0.9% saline with final DMSO concentration at 20% (v/v). Groups of mice (P2-P8) received the drug through intraperitoneal (IP) injection at 30 mg/kg body weight (kgBW) per day.

### *In Silico* target validation

We mined the Kyoto Encyclopedia of Genes and Genomes (KEGG) database, to find those mTOR pathway proteins involved in biogenesis and cell growth^29,30^. ALS-associated proteins were downloaded from 「Gene report of major ALS genes」 in the Amyotrophic Lateral Sclerosis Online Database (ALSoD)^27^. The two lists were then loaded into STRING, a protein-protein interaction database^28^ to search for functional protein associations. Those proteins involved in 「neuron projection」 [annotation from gene ontology (GO) database]^32^ were highlighted in the interactome and considered as enriched proteins in neurons. The proteins of the mTOR pathway with physical interactions toward ALS-associated proteins were considered as potential candidates for pharmacological manipulation of cell size.

### Two-Photon imaging and analysis

#### Spinal cord preparation

For two-photon fluorescent imaging, P8-P10 mice were deeply anesthetized with isoflurane and then supplied oxygen through face mask (95% O_2_−5% CO_2_). The spinal column was exposed surgically at the thorax and oxygenated, modified artificial cerebrospinal fluid (mACSF) containing (mM): NaCl, 126.0; KCl, 3.0; NaH_2_PO4, 1.0; MgSO_4_ ‧7H2O, 1.5; CaCl_2_ ‧2H2O, 2.5; NaHCO_3_, 26.2; and glucose, 10.0; with pH7.4, osmolality 300–305 mOsm/kg, room temperature; flowing at 5–7 ml/min was used to superfuse the spinal cord. The spinal column was then carefully removed from thoracic to sacral segments with continuous spinal cord superfusion. The dura was then removed, followed by decapitation and spinal cord transection above lumbar section. The ventral and dorsal roots were trimmed from the spinal foramen. The lumbar spinal cord with attached roots was then transferred to a petri dish filled with mACSF and bubbled with 95% O_2_–5% CO_2_ where any remaining dura mater and debris were gently removed and the spinal roots were trimmed to optimal length. After 30 minutes incubation at room temperature, the lumbar spinal cord was transferred to and fixed in a superfusion chamber, ventral side up. Finally, the spinal cord was positioned on the microscope stage for two-photon scanning with 95% O_2_–5% CO_2_ oxygenated mACSF superfusion (5–7 ml/min) throughout the scanning.

#### Two-photon imaging and analysis

P2-P7 neonates were administrated with vehicle or PF-4708671 at 30 mg/kg per day through IP injection and euthanized at P8-P10 as described previously. The lumbar spinal cord was scanned by DIC-fluorescent microscopy (Ultima LSM, Prairie, WI) equipped with mercury fluorescence light source (Olympus, Center Valley, PA) and Ti-Sapphire laser (Chameleon Ultra I, Coherent, CA) tuned at 920 nm. The imaging depth was extended to approximately 200 μm to include as much detail as possible, and was focused on ventrolateral motoneuron pools which could be easily identified by cell morphology and the density of GFP-positive neurons. The scanned images were reconstructed and analyzed in Python to measure motoneuron soma volume. Only GFP-positive neurons with maximum soma diameters > 20 μm with a thick, apical dendritic arbor projecting toward the pia mater and typical pyramidal shape were deemed to be motoneurons. On average, 5 motoneurons per animal that met these criteria and had clear boundaries were selected for reconstruction and further analysis. The total number of cells and animals per group is provided in the legend for Fig. 2. The experimenters were partially blinded to the specimen's grouping in that the tissue was sent out for genotyping after the imaging, and its group was revealed following the completion of image analysis.

### Whole-Cell patch clamp and analysis

#### Lumbar spinal cord slice preparation

Whole-cell patch-clamp recordings were made from spinal cord slices derived from P7-P9 mice. To prepare the slices, the cleaned spinal cord was quickly embedded in a semifluid agarose gel at −20 °C. Once the gel set, a cuboid of the embedded spinal cord was glued onto a pre-chilled metal specimen tray of Vibratome 1000plus (Leica, Buffalo Grove, IL, USA) with an ice bed beneath. The specimen tray was then filled with ice-cold, oxygenated dissecting solution. The Vibratome was set at: 7.5/10 amplitude (1.5 mm at maximal); 60 Hz; 100 μm/s speed (slowest); 20 ° blade angle and transverse, 350 μm slices were cut. The lumbar spinal slices were transferred by soft brush to an incubation chamber and maintained at 30 °C–32 °C with an 『incubating' ACSF containing (mM) NaCl, 126.0; KCl, 2.5; CaCl_2_ ‧2H_2_O, 2.0; MgCl_2_ ‧6H_2_O, 2.0; NaHCO_3_, 26.0; and glucose, 10.0; pH 7.4 when bubbled with 95% O_2_–5% CO_2_. After >1.5 hr incubation, a cord slice was loaded onto the stage of a differential inference contrast-infrared (DIC-IR) microscopy system (Eclipse E600FN, Nikon, Melville, NY) and perfused at 2.5 ml/min with oxygenated modified Ringer’s solution containing (mM): NaCl, 111; KCl, 3.09; NaHCO_3_, 25.0; KH_2_PO_4_, 1.10; MgSO_4_ ‧ 7H_2_O, 1.26; CaCl_2_ ‧ 2H_2_O, 2.52; and glucose, 11.1; room temperature.

Whole-cell patch-clamp electrical recordings were made as described by Quinlan *et al*.^12^ with an internal solution containing (mM): potassium gluconate, 138; HEPES, 10; ATP-Mg, 5; and GTP-Li, 0.3; by gauge 34 Microfil syringe (World Precision Institute) attached with 0.22μm filter (Millipore, Billerica, MA, USA). Pressure in the system was monitored by pressure manometer (World Precision Instrument). Recordings were made from neurons in the ventrolateral motoneuron pools characterized by their large soma size (>20 μm) and pyramidal shape. Only one motoneuron was impaled in each slice.

#### Protocols for PIC and F-I relationship

The recordings were controlled by pClamp 10 (Molecular Devices). Liquid junction potentials were corrected with pClamp 10 built-in function as soon as glass pipette (3–5 MΩ) was lowered into solution. In current-clamp mode, membrane potential was continuously recorded for 30 s following membrane rupture. Subsequently, in voltage-clamp mode, symmetrical triangular ramps commands from −80 mV to + 10 mv and back in 9 s were applied. A series of small voltage steps were used to estimate conductance and leak current. Then returning to current-clamp mode, symmetrical depolarizing current ramps at 600 pA/s, reaching a peak of 1,500 pA were injected into the cell. The current ramp recordings were used to calculate the cell's current threshold (I_on_), current offset (I_off_), the difference between I_on_ and I_off_ (ΔI), and frequency-current (F-I) relationship. The rheobase was also measured based on a series of depolarizing current steps.

#### Neuron selection and data analysis

Data analyses were carried in Clampfit (Molecular Devices) and Python. Only motoneurons with soma diameters >20 μm, resting membrane potentials < −50 mV, action potential peaks >0 mV, input resistances <110 M Ω and series resistances <25 M Ω were included in our sample of putative motoneurons. Further, cells whose resting membrane potentials or series resistances varied more than 10 mV or 10 M Ω during the recording session were excluded. The experimenters were partially blinded, as the tissues were sent out for genotyping after the recordings were completed and the genotyping result was only revealed upon completion of data analysis.

### Statistical analysis

Means ± standard deviations (SD) of all parameter values were calculated and box plot were used to present data range. One-way and two-way ANOVA were performed for multiple comparisons between groups and repeated measurements. Post-hoc pairwise comparison (Tukey's method) was further used to determine which group differences were statistically significant. Only P value <0.05 was considered significant. Statistical analyses were performed using SPSS (IBM, Armonk, NY, USA) and Microsoft Excel software applications (Redmond, WA, USA).

## Supplementary information

Supplementary Figures.

## Data Availability

The datasets generated during and/or analyzed during the current study are available from the corresponding author on reasonable request.
