# Supplementary material for: Excessive Homeostatic Gain in Spinal Motoneurons in a Mouse Model of Amyotrophic Lateral Sclerosis
Source: Sci Rep. 2020 Jun 3;10:9049. doi: 10.1038/s41598-020-65685-8 (PMC7271238; doi:10.1038/s41598-020-65685-8)
Supplement: Supplementary file 1 — Supplementary Figures. [file 41598_2020_65685_MOESM1_ESM.docx]

**Supplementary Data**

**Excessive Homeostatic Gain in Spinal Motoneurons in a Mouse Model of Amyotrophic Lateral Sclerosis**

Su-Wei Kuo^a^, Marc D. Binder^b^ and C.J. Heckman^a,c,d^*

a Department of Physiology, Northwestern University, Chicago, IL 60611, USA

b Department of Physiology & Biophysics, University of Washington School of Medicine, Seattle, WA 98195, USA

c Department of Physical Medicine and Rehabilitation, Northwestern University, Chicago, IL 60611, USA

d Department of Physical Therapy and Human Movement Sciences, Northwestern University, Chicago, IL 60611, USA

*Correspondence to CJ Heckman, 303 E. Chicago Ave, Ward 5-334, Northwestern University, IL 60611, USA.

Email: [c-heckman@northwestern.edu](mailto:c-heckman@northwestern.edu)

Tel: 312-503-2164

**S1. Optimization of PF-4708671 dosage**

PF-4708671 dosage was optimized by a toxicology test conducted on P2-P8 mice with daily IP injections of PF-4708671 in 10 mg/kg (N=13), 30 mg/kg (N=16) and 60 mg/kg (N=14) over a period of 6 days. Littermate controls received equivalent vehicle injections. The 60 mg/kg per day group showed a significantly increased mortality rate starting from P4 and a much lower overall survival rate at P8 (P=0.001). In contrast, the 10 mg/kg and 30 mg/kg per day groups and the vehicle control group showed no change in mortality rate. These results led us to test the efficacy of the 30 mg/kg per day dose of PF-4708671 on regulating cell size.

(S2A) (S2B)


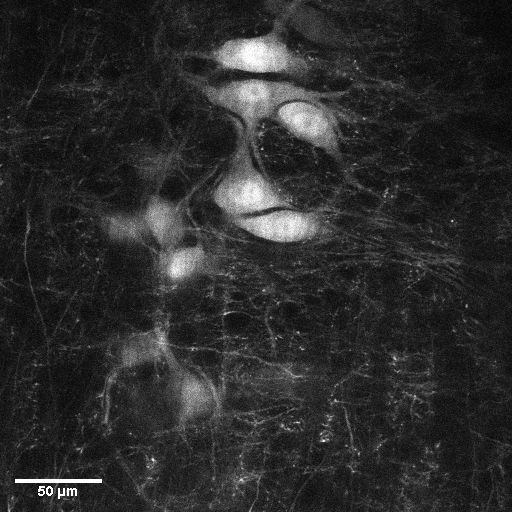

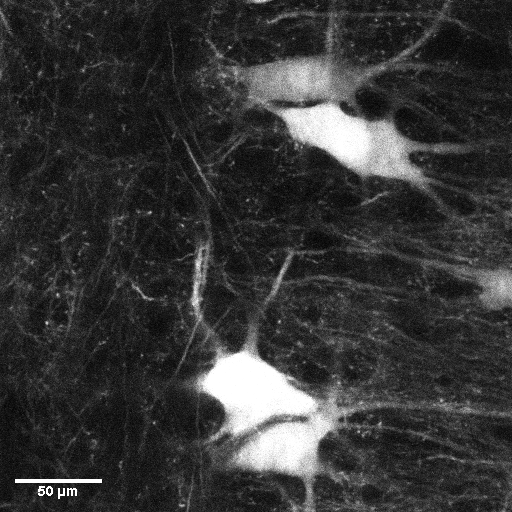


(S2C) (S2D)


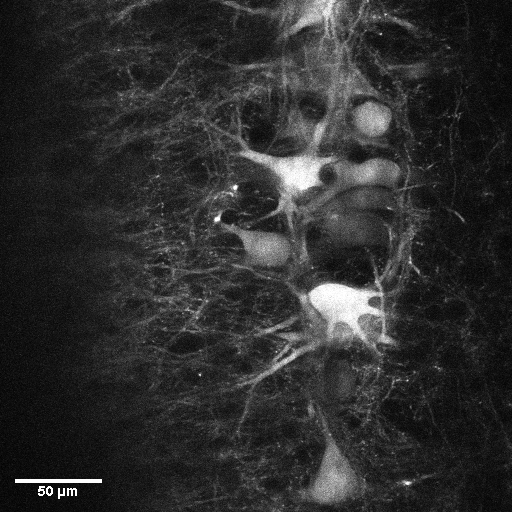

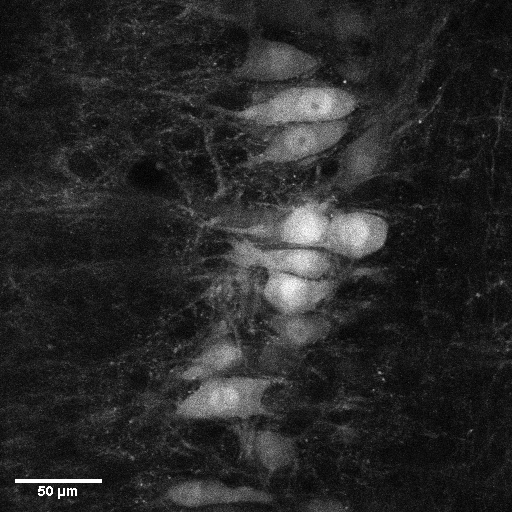


**S2. Representative image from two-photon microscopy**

**A**, Wild-type motoneurons subjected to two-photon scanning (40x) from ventrolateral spinal cord tissue. **B**, Certain **hG93A-SOD1 motoneurons showed increased cell size, which shifted the overall distribution curve as shown in Figure 2B. C** and **D**, Wild-type and **hG93A-SOD1 motoneurons received PF-4708671 have cell size distributed in similar range.**
